# Supplementary material for: Patterns of Intron Gain and Loss in Fungi
Source: PLoS Biol. 2004 Nov 30;2(12):e422. doi: 10.1371/journal.pbio.0020422 (PMC532390; doi:10.1371/journal.pbio.0020422)
Supplement: Table S1 — Also available at http://genes.mit.edu/NielsenEtAl/. (4.3 MB ZIP). [file pbio.0020422.st001.zip › NielsenEtAl/html/1405.html]

AN5713.1.NCU09700.1.MG03310.1.FG06306.1


```
 CLUSTAL W (1.82) Multiple Sequence Alignments - Introns Inserted


Sequence 1: NCU09700.1	557 aa
Sequence 2: MG03310.1	558 aa
Sequence 3: FG06306.1	556 aa
Sequence 4: AN5713.1	563 aa
Alignment Length: 564 aa
Number Identitical Residues: 433 aa
Alignment Score (without introns) 17735


MG03310.1 	MSFGGQTPTIIVLKEG1TDSSQGKGQIVSNINACLAVQATIKSTLGPYGGDLLMVDANGK
NCU09700.1	MSFGGQTPTIIVLKEG1TDTSQGKPQIISNINACLAVQATIKSTLGPYGGDLLMVDANGK
FG06306.1 	MSFGGQAPTIIVLKEG1TDTSQGKGQIISNINACLAVQATIKSTLGPYGGDLLMVDENGR
AN5713.1  	MAFAGQTPTIVVLREG1TDASQGKGQILSNINACVAVQSTVKSTLGPYGGDLLLVDSNGK
          	*:*.**:***:**:** **:**** **:******:***:*:************:** **:

MG03310.1 	QTITNDGATVMK0LLDIVHPAARVLVDIARSQDAEVGDGTTSVVVLAGEILKEIKEHVEQ
NCU09700.1	QTITNDGATVMK~LLDIVHPAARILVDIARSQDAEVGDGTTSVVVLAGEILKEVKEHVEQ
FG06306.1 	QTITNDGATVMK0LLDIVHPAARILVDIARSQDAEVGDGTTSVVVLAGEILKEVKEHVEQ
AN5713.1  	QTITNDGATVMK0LLDIVHPAARILTDIARSQDAEVGDGTTSVVVLAGEILKEVRELVEQ
          	************ **********:*.***************************::* ***

MG03310.1 	GVSSQIIVKGLRRASSVAVNKIKEIAVSTSEGS-----HRETLTKLAGTAMTSKLIKRNT
NCU09700.1	GVSSQIIIKGLRRASTMAVNKIKEIAINTAESN-----RRETLSKLAATAMTSKLIKRNT
FG06306.1 	GVSSQIIIKGLRRASQMAVNKIKEIAVSTNEAN-----QRDTLIKLAGTAMTSKLIKRNT
AN5713.1  	GVSAQTIVKGLRTSSAMAVNKVKEIAVDMIDAAGSEEKKIETLRRLAATAMNSKLIKRNS
          	***:* *:**** :* :****:****:.  :. .:...: :** :**.***.*******:

MG03310.1 	EFFTKM1VVDAVLTLDQEDLNEKLIGIKKIPGGSLTDSLFVNGVAFKKTFSYAGFEQQPK
NCU09700.1	DFFTEM1VVDAVLSLDQEDLNEKLIGIKKIPGGSLTDSLFVNGVAFKKTFSYAGFEQQPK
FG06306.1 	TFFTKM1VVDAVLSLDQDDLNEKLIGMKKIPGGSLTDSLFVNGVAFKKTFSYAGFEQQPK
AN5713.1  	DFFTKM1VVDAVLSLDQDDLNEKLIGVKKVTGGGLQDSLFVNGVAFKKTFSYAGFEQQPK
          	 ***:* ******:***:********:**:.**.* ************************

MG03310.1 	SFKKPKIVCLNVELELKAEKDNAEVRVEQVSEYQAIVDAEWQIIFKKLEAVYKTGAKVVL
NCU09700.1	SFKNPKICCLNVELELKAEKDNAEVRVEQVSEYQAIVDAEWQIIYKKLEAVYATGAKVVL
FG06306.1 	SFDDPKIVCLNVELELKAEKDNAEVRVEQVSEYQAIVDAEWQIIYKKLDAIHKTGAKVVL
AN5713.1  	SFKNPKIVCLNVELELKSEKDNAEVRVEQVSEYQAIVDAEWQIIYNKLEAIYKSGAKVVL
          	**..*** *********:**************************::**:*:: :******

MG03310.1 	SKLPIGDLATQ~YFADRDIFCAGRVASEDMERVVQATGATVQSTCSDILPEHLGTCGSFE
NCU09700.1	SKLPIGDLATQ~YFADRDIFCAGRVTSEDMERVVQATGATVQSTCSDILPEHLGTCGTFE
FG06306.1 	SKLPIGDLATQ~FFADRDIFCAGRVAAEDMERVVQATGAVVQSTCSDILHEHLGTCGKFE
AN5713.1  	SKLPIGDLATQ2YFADRDVFCAGRVASDDMDRVCQATGAAVQSTCSDIQDRHLGTCGSFE
          	*********** :*****:******:::**:** *****.********  .******.**

MG03310.1 	ERQIGGERFNFFEDCPEAKTCTLVLRGGAEQFIAEVERSLHDAIMIVKRAIRNRTVVAGG
NCU09700.1	ERQIGGERFNFFEDCPSAKTCTLVLRGGAEQFIAEVERSLHDAIMIVKRAIRNKTIVGGG
FG06306.1 	ERQIGGERFNFFENCPEAKTCTLVLRGGAEQFIAEVERSLHDAIMIVKRAIRNQLIVGGG
AN5713.1  	ERQIGGERYNLFSECPKAKTCTLVLRGGAEQFIAEVERSLHDAIMIVKRALRNTTIVAGG
          	********:*:*.:**.*********************************:**  :*.**

MG03310.1 	GAVEMEVSAHLHRFADLKNQAHKQQAIIKSFAKALEIIPRQLCDNAGFDATDILNKLRVE
NCU09700.1	GAVEMEVSAYLHRFAD-RDVAHKQQAIIKSFAKALEIIPRQLCDNAGFDATDILNKLRVE
FG06306.1 	GAVEMEISAYLHQFAD-KNIPHKQQAIIKSFAKALEIIPRQLCDNAGFDATDILNKLRVE
AN5713.1  	GATELELSGYLHRYAD-RNVPYKQQAVVKAFAKALEVIPRQLCDNAGFDATDILNRLRVE
          	**.*:*:*.:**::** :: .:****::*:******:******************:****

MG03310.1 	HRKGNTWAGVDFQNEGVADLMERFVWEPALIKINAIQAATEACCLILSVDETIR~NEESA
NCU09700.1	HRKGNTWAGVDFTNEGVCDMMERFVWEPALIKINALQAATEAACLILGVDETIR~NEESQ
FG06306.1 	HRKGQTWAGVDFQNEGVTDMMERFVWEPALVKVNALQAATEASCLILGVDETIR~NEESA
AN5713.1  	HHKGNTWAGVDFDHEGVRDNMAAFVWEPSLVKVNAIQAAVEAACLILSVDETIK1NQESA
          	*:**:******* :*** * *  *****:*:*:**:***.**.****.*****: *:** 

MG03310.1 	QPQAPGQALPPGAAQRALRG-RGRGMPRR
NCU09700.1	APQAPGQKLPPGAAQRALRG-RGRGMPRR
FG06306.1 	KPQAPGQ-LPPGAAQRALRG-RGRGMPRR
AN5713.1  	APQAPSRGLPPGAAQRALGGGRGRGMPRR
          	 ****.: ********** *.********
```
